# The Atlantic Monthly

**Published:** 1867-02

**Authors:** 


The Atlantic Monthly.—The Atlantic Monthly enters on its nineteenth volume with an array of distinguished names and sterling articles that promise well for the coming year. The January number contains the first instalment of Dr. Holmes's story, "The Guardian Angel," in which will be found the same old charm that so fascinated the readers of the *Autocrat*, the *Professor*, and *Elsie Venner;* a humorous story in verse, by James Russell Lowell; a graphic sketch of Henry Ward Beecher's church, with some pertinent reflections upon modern church-going, by James Parton; a legend in verse, told as only Whittier can tell it; a poem entitled "Terminus," (on Growing Old,) by R. W. Emerson; a spirited and faithful translation of the contest between Achilles and Agamemnon, from the First Book of the Iliad, by W. C. Bryant. Mr. Higginson contributes a Plea for culture; Mr. Trowbridge furnishes another of his attractive stories under the title, The Man who stole a Meeting House; Bayard Taylor tells a characteristic story of The Strange Friend; Mr. Shanly gives a humerous sketch of Capilary Freaks; E. C. Stedman offers a poem on Pan in Wall Street; and Walter Mitchell describes the Kingdom of Infancy. The story of Katharine Morne, by the author of "Herman," is continued. Topics of current political interest are thoroughly treated—the Causes for which a President can be Impeached are lucidly set forth, and Frederick Douglass makes a powerful Appeal to Congress for Impartial Suffrage. The number closes with notices of several popular new publications.

